# Trends in Incidence of Hepatocellular Carcinoma Between 2000 and 2020: A Surveillance, Epidemiology, and End Results (SEER)-Based Analysis

**DOI:** 10.7759/cureus.84487

**Published:** 2025-05-20

**Authors:** Urmimala Chaudhuri, Chase T Thornton, Timothy N Crawford, Drew Triplett

**Affiliations:** 1 Internal Medicine Residency Program, Wright State University, Dayton, USA; 2 Internal Medicine, Wright State University, Dayton, USA; 3 Public Health, Wright State University, Dayton, USA; 4 Gastroenterology and Hepatology, Wright State University, Dayton, USA

**Keywords:** cancer incidence, epidemiology and public health, health care disparities, health surveillance, liver cancer

## Abstract

Introduction

Hepatocellular carcinoma (HCC) remains a significant global health concern, ranking as the third leading cause of cancer-related mortality worldwide. The risk factors for HCC include chronic hepatitis B and C, alcohol-associated liver disease, and non-alcoholic fatty liver disease (NAFLD). The aim of this study was to evaluate trends in HCC incidence in the United States from 2000 to 2020 using the Surveillance, Epidemiology, and End Results (SEER) database, with stratification by age, sex, and race/ethnicity to identify demographic differences in temporal patterns.

Methods

Incidence rates of HCC per 100,000 population (age-adjusted) from 2000 to 2020 were calculated utilizing a population-based cancer registry, SEER*Stat (version 8.4.3). HCC cases were identified by the International Classification of Diseases for Oncology, third edition (ICD-O-3) site code C22.0 and histology codes 8170-8175, including only histologically confirmed malignancies. We utilized the Joinpoint Regression Program version 5.0.1 to report time-trends expressed as annual percentage change (APC). Cases with missing age, sex, or race data were excluded from stratified analyses. Statistical significance was set at a p-value less than 0.05.

Results

There were a total of 242,769 cases of HCC recorded from 2000 to 2020. Since 2015, HCC incidence has declined across all age groups, with the most significant reductions observed in individuals aged 45-54 years (APC, −8.22; p < 0.05) and 55-64 years (APC, −7.03%; p < 0.05). Among racial and ethnic groups, Black individuals experienced the most pronounced decline between 2018 and 2020 (APC, −10.64%; p < 0.05), followed by Hispanics (APC, −8.25%; p < 0.05) and Asians (APC, −8.06%; p < 0.05). With regards to sex, there was a greater decline among males (APC, −7.28%; p < 0.05) from 2018 to 2020 compared to females (APC, −2.4%; p < 0.05) from 2014 to 2020.

Conclusions

These findings reflect encouraging trends likely due to improved prevention and treatment of chronic liver diseases and are consistent with declining HCC incidence observed in other high-income countries. However, reliance on histologically confirmed diagnoses may underestimate the true incidence. Persistent disparities, potentially driven by differences in access to care and socioeconomic status, indicate a need for targeted interventions addressing underlying risk factors of HCC, such as obesity, alcohol, and viral hepatitis. With continued efforts focused on prevention, early detection, and treatment, the burden of HCC will continue to decline.

## Introduction

Primary liver cancer is the sixth most diagnosed malignancy and the third leading cause of cancer death worldwide [1]. Although most cases occur in East Asia and Africa, accounting for up to 72% of the global burden, approximately 5% of cases occur in the United States [1]. Hepatocellular carcinoma (HCC) constitutes over 90% of primary liver cancers, with more than 70% historically linked to chronic hepatitis B and C infections [2]. Additional risk factors include alcohol-related liver disease and non-alcoholic fatty liver disease (NAFLD), the latter of which is rapidly increasing in prevalence, especially in Western countries [3]. It is estimated that 19.2% of HCC cases are due to NAFLD in the United States [4]. On the contrary, about 32-45% of HCC cases are related to alcohol [5].

While HCC is often associated with modifiable risk factors such as viral hepatitis, alcohol use, and obesity, its prevention is complex and influenced by genetic predisposition, socioeconomic conditions, and healthcare access. Although effective strategies such as hepatitis B vaccinations, antiviral therapies, and lifestyle interventions are available, they are underutilized. Compounding this issue is the lack of widespread screening programs for high-risk populations, leading to late-stage diagnoses due to the typically asymptomatic early course of HCC [6]. Additionally, the Surveillance, Epidemiology, and End Results (SEER) database, which is nationally representative in scope, includes select geographic regions and may underrepresent certain populations, which should be considered when interpreting these findings. 

The epidemiologic landscape of HCC is shifting. While the incidence of viral hepatitis-related HCC is declining due to vaccination and curative antiviral therapies, non-viral causes such as NAFLD and alcohol-related liver disease are becoming more prominent. These shifts underscore the need for updated trend analysis that reflects the changing risk factor patterns, demographic dynamics, and the impact of public health measures. 

Our study aimed to examine national trends in HCC incidence in the United States from 2000 to 2020 using the SEER database. This period captures major changes in liver disease patterns, public health interventions, and access to antiviral therapies. We further stratified incidence trends by age, sex, and race to better understand the evolving burden and guide targeted prevention strategies.

## Materials and methods

Study design and study population

This was a retrospective, population-based cohort study using the SEER program of the National Cancer Institute. Data was extracted from SEER 22 registries, which cover approximately 28% of the U.S. population and are known for high data quality, standardized coding, and comprehensive case capture. To ensure consistency and comparability, only cases from registries available across the entire study period were included in the trend analysis. We used SEER*Stat software (version 8.4.3) to generate age-adjusted incidence rates of HCC from 2000 to 2020. 

Study population and case selection

Patients with a diagnosis of HCC were identified using the International Classification of Diseases for Oncology, third edition (ICD-O-3). Eligible cases included those with the primary liver site code C22.0, with malignant behavior only. Histology codes were 8170-8175, indicating HCC. To ensure diagnostic accuracy, only microscopically confirmed malignant tumors were included. Only the first primary liver cancer cases were analyzed to avoid duplication from multifocal or recurrent tumors. Cases with unknown histology were excluded. While this approach improves specificity, it may underestimate the true incidence of HCC, particularly in patients who were not biopsy candidates due to advanced disease or comorbidities. We restricted the inclusion criteria to primary liver cancers diagnosed in adults aged greater than or equal to 20 years or older at the time of diagnosis, as HCC is extremely rare in pediatric and adolescent populations. The stratified analysis focused on age groups of 45 years and older due to the very low incidence in younger age brackets. 

Variables and stratification

We extracted incidence data stratified by sex (male, female), age group at diagnosis (45-54, 55-64, 65-74, and 75+), and race/ethnicity (non-Hispanic White, non-Hispanic Black, non-Hispanic Asian/Pacific Islander, non-Hispanic American Indian/Alaska Native, and Hispanic). Individuals were classified as Hispanic regardless of race, and those with unknown or “other” racial/ethnic identification were excluded due to small and heterogeneous sample sizes. These stratifications were selected to reflect established epidemiologic differences in HCC burden and facilitate evaluation of disparities across demographic subgroups. Incidence rates were age-adjusted to the 2000 U.S. standard population and expressed per 100,000 person-years. 

Statistical analysis

Trends in HCC incidence from 2000 to 2020 were analyzed using the Joinpoint Regression Program (version 5.1.0, National Cancer Institute). This software fits a series of joined log-linear segments to the data to detect statistically significant changes in incidence trends over time. It was chosen for its ability to estimate annual percentage changes (APCs) and identify inflection points, which are particularly relevant in long-term cancer surveillance. The analysis was stratified by sex, age group, and race/ethnicity. The model used log-transformed age-adjusted rates. The Monte Carlo Permutation method was used to test for statistical significance. The number of joinpoints was determined using the Joinpoint Regression Program’s default model selection method, which utilizes a permutation test based on the Bayesian Information Criterion (BIC) to identify the best-fitting model with a maximum of three joinpoints, as permitted by the number of time points (n = 21). Annual percent change (APC) was calculated for each segment, along with 95% confidence intervals. A p-value of <0.05 was considered statistically significant. Cases with missing data on age, sex, or race were excluded from stratified analysis but accounted for less than 1% of the dataset, minimizing potential bias. 

Ethical considerations

This study used publicly available and fully de-identified data from the SEER program. As such, it met the criteria for exemption from institutional review board (IRB) approval, and informed consent was not required.

## Results

A total of 242,769 HCC cases were recorded in the SEER database between 2000 and 2020. When stratified by sex, males had a higher age-adjusted incidence rate compared to females (45 vs. 10.4 per 100,000 person-years). The incidence rate of HCC increased with age, peaking in the 75-84 age group, followed by the 70-74 and 65-69 age brackets. HCC incidence also varied by race: 9.1 per 100,000 in non-Hispanic Whites, 12.7 in non-Hispanic Blacks, 10.7 in non-Hispanic American Indians, and 16.9 in non-Hispanic Asians. 

Nationally, the incidence of HCC rose steadily from 7.0 per 100,000 in 2000 to a peak of 11.5 in 2014, after which it plateaued and began to decline, reaching 9.4 by 2020. Joinpoint regression confirmed this transition, identifying significant shifts in incidence trends between 2014 and 2016 across most demographic groups (Table 1).

**Table 1:** Age-adjusted incidence rates of hepatocellular carcinoma (HCC) by sex, age group, race/ethnicity, and year in the United States from 2000 to 2020, based on Surveillance, Epidemiology, and End Results (SEER) data Rates are expressed per 100,000 person-years.

Category	Cases	Rate
Overall	242,769	10.1
Sex		
Male	179,396	45
Female	63,373	10.4
Age		
20-24 years	385	0.2
25-29 years	597	0.3
30-34 years	909	0.4
35-39 years	1,659	0.8
40-44 years	3,850	1.8
45-49 years	10,790	5
50-54 years	23,717	11.5
55-59 years	37,815	20.4
60-64 years	41,309	26.8
65-69 years	36,606	29.9
70-74 years	28,799	30.3
75-79 years	23,474	32.3
80-84 years	16,958	32
85+ years	13,565	26.2
Race		
White	175,384	9.1
Black	32,501	12.7
American Indian/Alaska Native	2,217	10.7
Asian or Pacific Islander	29,004	16.9
Year		
2000	6,308	7
2001	6,881	7.5
2002	7,277	7.8
2003	7,580	8
2004	8,351	8.6
2005	9,007	9.1
2006	9,433	9.3
2007	10,213	9.8
2008	10,706	10
2009	11,651	10.6
2010	11,752	10.4
2011	12,423	10.7
2012	13,181	11
2013	13,730	11.2
2014	14,526	11.5
2015	14,747	11.4
2016	14,823	11.2
2017	14,799	11
2018	14,828	10.9
2019	14,977	10.8
2020	13,240	9.4

Age-stratified trends

In the 45-54 age group, the APC was 7.9 from 2000 to 2005 (p < 0.05), followed by a non-significant decline from 2005 to 2012, and a significant decrease from 2012 to 2020 (APC, −8.22%; p < 0.05). Among individuals aged 55-64, incidence increased significantly from 2000 to 2009 (APC, 8.65%; p < 0.05), then stabilized, and significantly declined from 2014 to 2020 (APC, −7.03%; p < 0.05). In the 65-74 age group, incidence rose from 2000 to 2018. Similarly, in those aged 75+, incidence increased from 2000 to 2013 (APC, 2.95%; p < 0.05) but did not show a significant decline thereafter. Overall, the 45-54 and 55-64 age groups showed statistically significant declines in recent years. 

Race-stratified trends 

Among non-Hispanic American Indians, incidence increased from 2000 to 2016 (APC, 4.57%) and declined from 2016 to 2020 (APC, −7.24%), though neither trend was statistically significant. In contrast, non-Hispanic Asians experienced significant declines from 2007 to 2016 (APC, −2.19%; p < 0.05) and from 2016 to 2020 (APC, −8.06%; p < 0.05). For non-Hispanic Whites, incidence rose from 2000 to 2009 (APC, 4.40%; p < 0.05), slowed between 2009 and 2015 (APC, 2.01%; p < 0.05), and then significantly declined from 2015 to 2020 (APC, −2.25%; p < 0.05). Among non-Hispanic Blacks, APCs from 2000 to 2014 were positive but not statistically significant. However, from 2018 to 2020, a significant decline was noted (APC, −10.64%; p < 0.05). Among Hispanics, incidence declined in the most recent years (APC, −8.25%; p < 0.05). Overall, significant recent declines were noted in most racial groups, particularly among non-Hispanic Whites, non-Hispanic Asians, and non-Hispanic Blacks (Table 2). 

**Table 2:** Annual percent change (APC) in hepatocellular carcinoma (HCC) incidence by sex, age group, and race/ethnicity in the United States, 2000-2020, using Joinpoint regression analysis of Surveillance, Epidemiology, and End Results (SEER) data Asterisks (*) indicate statistically significant trends at p < 0.05.

Cohort	Lower endpoint	Upper endpoint	APC	Lower CI	Upper CI	p-value
Sex						
Female	2000	2014	2.8378*	2.4549	3.3183	<0.000001
Female	2014	2020	-2.0807*	-3.5612	-0.9912	0.005339
Male	2000	2007	5.0234*	4.0521	7.09	0.000104
Male	2007	2014	2.0519*	1.2141	4.5006	0.032161
Male	2014	2018	-1.0213	-2.2946	1.6766	0.292995
Male	2018	2020	-7.2848*	-9.4862	-3.9779	0.000404
Age						
45-54	2000	2005	7.9161*	5.1371	14.2416	0.004293
45-54	2005	2012	-1.5445	-3.8859	0.5635	0.178703
45-54	2012	2020	-8.2172*	-10.5116	-6.8912	0.000026
55-64	2000	2009	8.6469*	7.473	14.4442	0.000159
55-64	2009	2014	5.3835	-6.847	6.9708	0.102969
55-64	2014	2020	-7.0275*	-8.8674	-5.2492	0.000014
65-74	2000	2018	3.3472*	3.03	4.6527	0.049068
65-74	2018	2020	-3.5052	-8.5229	2.4455	0.168575
75+	2000	2013	2.9540*	2.2913	4.4005	0.001206
75+	2013	2020	-0.2709	-3.2898	1.1286	0.820137
Race						
Non-Hispanic American Indian	2000	2016	4.5690*	3.3043	10.0804	0.384837
Non-Hispanic American Indian	2016	2020	-7.2361	-23.4236	0.9882	0.269758
Non-Hispanic Asian	2000	2007	0.8986	-0.2999	4.8771	0.537173
Non-Hispanic Asian	2007	2016	-2.1948*	-3.4777	-1.2416	0.013027
Non-Hispanic Asian	2016	2020	-8.0577*	-12.3539	-5.9474	0.000401
Non-Hispanic White	2000	2009	4.3991*	3.7798	6.4556	0.000098
Non-Hispanic White	2009	2015	2.0116*	0.1337	3.2208	0.02644
Non-Hispanic White	2015	2020	-2.2507*	-4.4772	-1.1188	0.022959
Non-Hispanic Black	2000	2008	5.1678	-0.2341	12.528	0.056019
Non-Hispanic Black	2008	2014	2.0749	-1.2421	10.022	0.393664
Non-Hispanic Black	2014	2018	-2.4797	-4.0253	3.947	0.235041
Non-Hispanic Black	2018	2020	-10.6409*	-16.1177	-4.6906	0.00294
Hispanic	2000	2010	3.3983*	2.6514	5.1939	0.016294
Hispanic	2010	2018	0.2138	-0.5729	1.2608	0.681906
Hispanic	2018	2020	-8.2517*	-12.3205	-4.5764	0.00374

Sex-stratified trends

Among females, the incidence increased from 2000 to 2014 (APC, 2.84%; p < 0.05), followed by a significant decline from 2014 to 2020 (APC, −2.08%; p < 0.05). Among males, incidence rose from 2000 to 2007 (APC, 5.02%; p < 0.05) and continued to rise more slowly from 2007 to 2014 (APC, 2.05%; p < 0.05). While there was a non-significant plateau between 2014 and 2018, a significant decline occurred from 2018 to 2020 (APC, −7.28%; p < 0.05).

Joinpoint regression identified one to three statistically significant joinpoints, depending on the demographic subgroup. For the overall population, a major joinpoint occurred in 2014, which marked a shift from increasing to decreasing HCC incidence. Among sex-stratified groups, males had joinpoints in 2007 and 2014, whereas females had a single joinpoint in 2014. There were also variations in joinpoints when stratified by race. Non-Hispanic Whites had joinpoints in 2009 and 2015, whereas non-Hispanic Asians had significant changes in 2007 and 2016. Overall, these inflection points indicate that the timing of HCC incidence shifts differed across demographic groups. 

Individuals identified as Hispanic were categorized as “Hispanic” regardless of race and were excluded from the four non-Hispanic racial groups to avoid overlapping cohorts. Individuals missing or with “other” racial identification were excluded due to small, heterogeneous samples.

## Discussion

Population-wide trends

Our analysis of the SEER database from the National Cancer Institute reveals a compelling trend: HCC incidence in the United States declined from 2000 to 2020. Decreases, both statistically significant and non-significant, were observed in all ages, races, and sexes. This decline is likely multifactorial. The prevalence of chronic viral hepatitis, alcohol-related liver disease, and NAFLD, the three primary drivers of HCC, may be decreasing. Alternatively, these conditions might still be prevalent, but their progression to HCC may be mitigated by alternative improvements, such as screening, treatment, or public health measures. The reality is likely a combination of both. Finally, these propositions assume that our data’s demonstrated decline represents real epidemiologic changes. Hyperinflation by potential artifacts, such as would be seen with reduced screening, diagnostic delays, or underreporting, is plausible.

Prior analysis of the SEER database has demonstrated similar trends of decreasing HCC incidence, and it has been noted that obesity and alcohol-related death rates were stable or worsened, respectively, during the time in which HCC incidence began to plateau in the early 2010s. These observations were thought to suggest that the observed decline in HCC incidence may be predominantly attributable to improved management of hepatitis B and C infections [7]. While establishing direct causality remains challenging, this interpretation is conceivable and supported by available information.

Another possible factor is that individuals with non-viral liver cirrhosis have a significantly lower risk of progression to HCC. For example, large-scale population studies in Denmark and Great Britain found that alcohol-associated cirrhosis patients had up to three-fold lower rates of progression to HCC compared to those with viral-related cirrhosis [8]. Thus, as obesity and alcohol become prevalent causes of liver disease in the United States, the overall incidence of HCC may paradoxically decline, as is now occurring.

Still, this shift has public health consequences: nearly 39% of American adults are now obese [9], and alcohol-related deaths have risen sharply over the past two decades [10]. Individuals with NAFLD are more likely to die from cardiovascular disease, hepatic disease, or other malignancies before HCC can develop, which, again, would contribute to the observed decline in HCC incidence [11]. Furthermore, among those who develop HCC, non-HCC complications of liver disease remain the primary cause of mortality, except in late-stage disease [12].

Finally, while the incidence of HCC is declining, the incidence of intrahepatic cholangiocarcinoma (ICC), another major form of primary liver cancer, has been increasing. This inverse trend is particularly intriguing given nearly identical overlap in their risk factors [11]. While certain conditions, such as primary biliary cholangitis and choledochal cysts, are more specifically associated with ICC [13], these alone do not adequately explain the discrepancy. One potential explanation involves epigenetic regulation: depending on specific molecular and cellular contexts, cirrhotic hepatocytes may undergo malignant transformation toward either HCC or ICC [9]. Although a detailed exploration is beyond the scope of this paper, this inverse trend is noted here to highlight complexity in liver carcinogenesis, acknowledge additional areas for research, and call into question simplified explanations for the declining rates of HCC in the United States.

Sex-based trends

**Figure 1:**
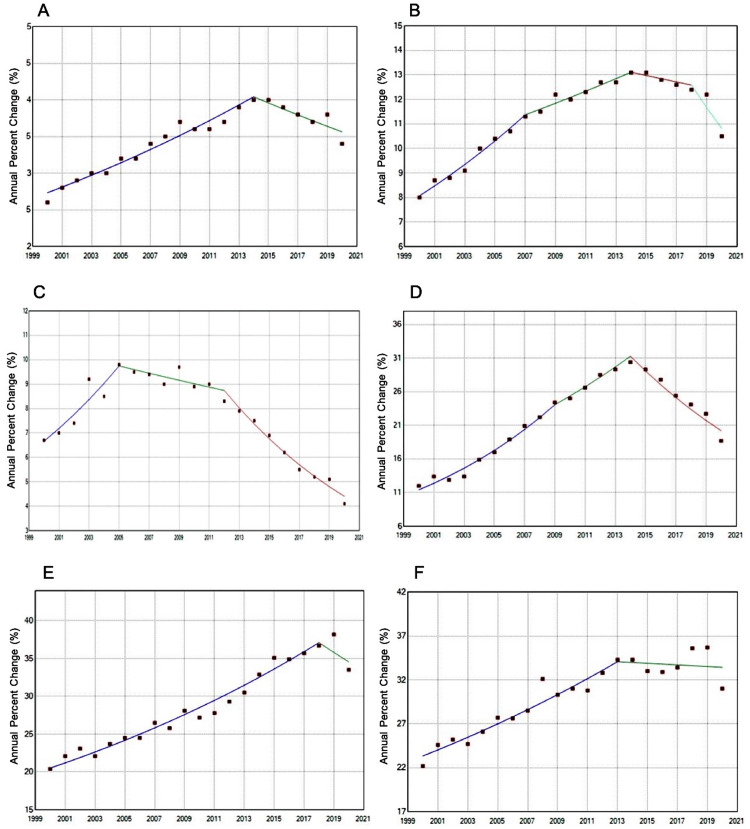
Age-adjusted rates of hepatocellular carcinoma by age and sex from Surveillance, Epidemiology, and End Results (SEER) 2000-2020 (A) Annual percentage change (APC) for females (B) APC for males (C) APC for age group 45-54 (D) APC for age group 55-64 (E) APC for age group 65-74 (F) APC for age group 75+

As consistently observed in the literature, HCC incidence remains lower among females than males [14]. Our data shows that this trend persists, but both sexes experience declining incidences. Notably, males had a steeper decline in recent years, with an APC of −7.28% from 2018 to 2020 (Figure 1). One large, multicenter study demonstrated notable differences in underlying etiology between sexes. Females had a higher frequency of NAFLD, while males had a higher frequency of alcohol-associated liver disease [15]. Therefore, the trend seen in our data could reflect improved alcohol use behaviors among males. However, this is merely a supposition. Aside from worse incidence, males are also more likely to be diagnosed at later stages of HCC, when curative treatments are less viable. This is partly due to lower adherence to surveillance guidelines by males, although biological differences may also contribute to more aggressive disease among males [16].

Age-based trends

While all age groups demonstrated decreasing HCC incidence, younger and middle-aged cohorts (ages 45-64) experienced more recent robust declines (APCs of −8.21% and −7.03%, respectively). In contrast, older adults (65+) showed smaller, statistically insignificant declines (APCs ranging from −3.5% to −0.27%) (Figure 1). This pattern is thought to reflect generational differences in exposure to hepatitis B and C, with younger or middle-aged cohorts experiencing lower rates of chronic hepatitis compared to prior generations.

This inference is based on data gathered by the United States over the past several decades. National Health and Nutrition Examination Surveys revealed that hepatitis B incidence among American children decreased substantially following the Centers for Disease Control and Prevention (CDC)’s 1991 recommendation for routine childhood vaccination in addition to international vaccine efforts [17]. From 1991 through 2011, acute hepatitis B cases in children declined. In 2022, hepatitis B vaccination guidelines expanded to include all adults aged 19-59, regardless of risk factors [18]. Similarly, the CDC found that the incidence of chronic hepatitis C has declined since 2016, supported by improved screening, treatment strategies, and behavioral changes. The CDC similarly adopted recommendations for universal adult hepatitis C screening in 2020 [19].

Race-based trends

**Figure 2:**
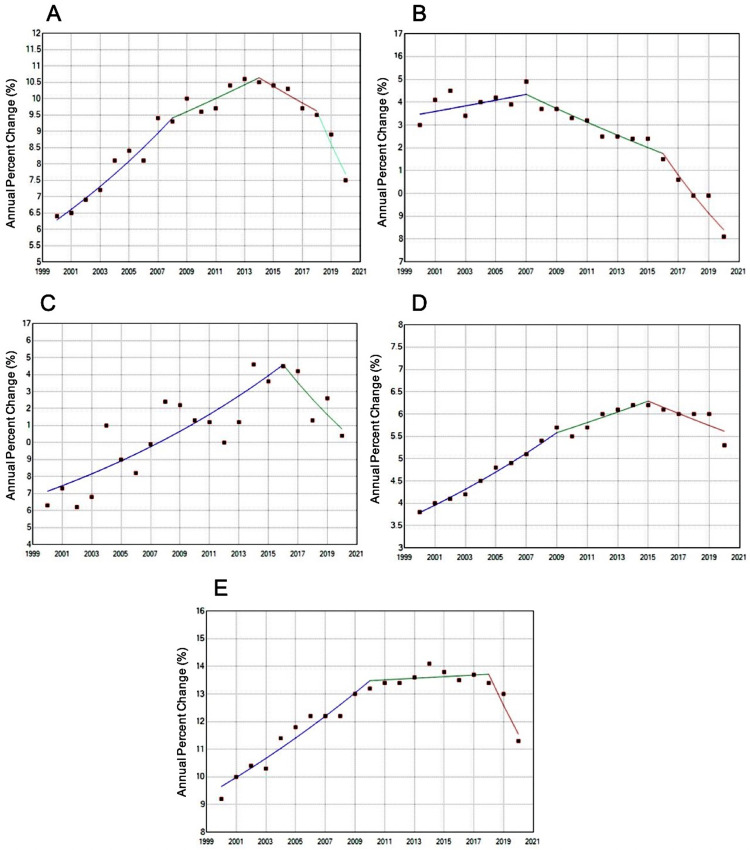
Age-adjusted rates of hepatocellular carcinoma by race from Surveillance, Epidemiology, and End Results (SEER) 2000-2020 (A) Annual percentage change (APC) for non-Hispanic Black (B) APC for non-Hispanic Asian (C) APC for non-Hispanic American Indian (D) APC for non-Hispanic White (E) APC for Hispanic

Our data reveals promising reductions in HCC incidence across racial groups, with non-Hispanic Asians and Hispanics demonstrating the most pronounced improvements. Non-Hispanic Asians, despite having the highest overall HCC incidence (16.9 per 100,000) over the 20-year period, experienced a statistically significant decline from 2007 to 2020, including a marked decline from 2016 to 2020 with an APC of −8.06%. Hispanics were the only other group to show a more statistically significant decline, with an APC of −8.25% from 2018 to 2020 (Figure 2).

It is unclear why prior racial disparities may be improving. Historically, higher incidence rates in Hispanics and Asians, specifically Indian subgroups, have been attributed to genetic predispositions [20]. However, the improvements observed across races in our study suggest other possibilities. Potential explanations include better access to care, more standardized screening, or broader implementation of hepatitis treatments, but one would not expect genetic predispositions to have changed over such a short time course. While past studies reported limited improvement among Blacks and American Indians [21], our findings suggest potential for decline in these groups, too, albeit not statistically significant.

Limitations

While the SEER database offers valuable population-level insights, it has limitations. Most notably, it lacks data on disease etiology, preventing us from definitively linking trends to underlying causes. Additionally, socioeconomic status and education level, factors associated with HCC risk, are not available in SEER, although the database does reflect national averages in these domains [22].

Fortunately, the association between education and socioeconomic status with the three major causes of HCC (obesity/metabolic syndrome, chronic hepatitis, alcohol use) has been studied. Obesity is notably tied to lower educational attainment and lower income levels [23-28]. While alcohol use presents a more complex picture, with high-risk behaviors linked both to lower education and, in certain contexts, higher wages, its role in HCC development remains clear [29-31].

Other sources of bias include patient migration, which can lead to incomplete survival or incidence tracking as individuals who move away from a SEER-participating region represent lost data points. SEER regions contain a higher proportion of foreign-born individuals, which, in theory, could impact incidence results [32]. Since hepatitis B prevalence is higher in certain countries, especially in parts of Africa where 5-10% of adults are chronically infected with hepatitis B [33], this could influence SEER data and overestimate U.S.-wide incidence rates. Finally, coding variability remains a potential source of error, though SEER actively works to minimize this issue [32].

**Figure 3:**
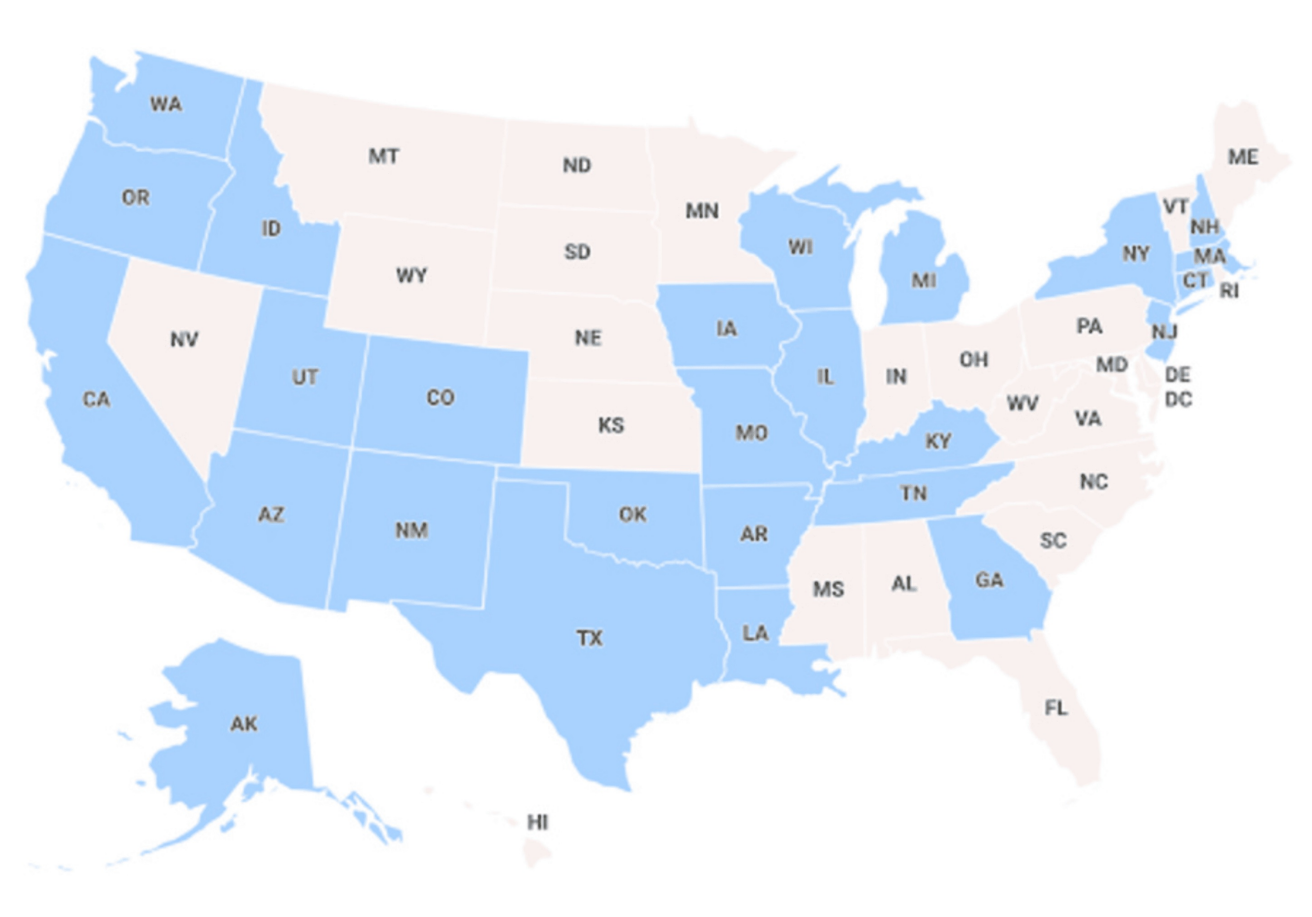
Surveillance, Epidemiology, and End Results (SEER) coverage map The states highlighted in blue are home to individuals participating in the SEER database.

Another source of bias involves barriers, with the incorporation of Native American-specific cancer information into the SEER database. The National Cancer Institute has been able to incorporate data from the New Mexico Tumor Registry (NMRT), Cherokee Nation Cancer Registry (CNCR), and Alaska Native Tumor Registry (ANTR) into the SEER database. Consequently, incidence trends of Native Americans/Alaska Natives are more reflective of certain tribes, while other tribes may be under-represented. Further, SEER assigns each individual into a single, broad racial group based upon what the individual most identifies with regarding race. This approach, although reasonable, is another limitation to note. Again, by the nature of collecting their data from multiple databases in order to form the SEER database, certain states or populations are not accounted for. For example, several Great Plains and the Mid-Atlantic states are not included. Finally, the potential for misclassification or underreporting is an inherent limitation of cancer registries, including the SEER database. Given that SEER integrates data from multiple sources, variability and inconsistencies in cancer-related coding within electronic medical records present a significant challenge to data accuracy. Despite these sources of sampling biases, the strength of SEER’s ever-expanding coverage should be acknowledged. SEER estimates a current coverage of approximately 45.9% of the United States, with the states covered included in Figure 3 [22]. There have been efforts to ensure quality data with coding standardization, etc. Despite inherent limitations, the data can provide useful insights.

Finally, a lack of multivariate analysis in our paper means our conclusions do not reflect the relationship between multiple variables. This could lead to inaccurate conclusions or a missed understanding of the complex mechanisms driving decreased HCC incidence. The conclusions drawn in our paper presume the potential for causal relationships that the observational nature of this paper cannot definitely establish. External data or research has been utilized to extrapolate information to support our inferences regarding observed trends. Some of the external data is strong and well-supportive, while other theories proposed in our discussion highlight areas in which further validation is needed. Despite these major limitations, value remains in our clear demonstration of statistically significant declines in incidence in several groups in the United States, even if the various mechanisms behind this decline are less plain.

Future Directions

Prevention and Public Health Implications

Despite the recent decline in HCC incidence, overall incidence rates in 2020 remain higher than those in 2000. This highlights the need for sustained and enhanced public health efforts. Our data indicates that HCC incidence begins to rise significantly after the age of 40 and continues to increase into older age groups. Targeted screening strategies, particularly for individuals with cirrhosis or other identifiable risk factors, could aid in early detection and improve survival outcomes. There is already evidence supporting the use of biannual hepatic ultrasound in cirrhotic patients, which has led to earlier detection and improved treatment outcomes. The American Association for the Study of Liver Diseases recently updated its screening guidelines to include at-risk populations including the following: 

o Child-Pugh A or B cirrhosis patients from any etiology

o Child-Pugh C cirrhosis patients who are transplant candidates

o Chronic hepatitis B patients regardless of cirrhosis status

Individuals with chronic hepatitis C (without advanced fibrosis) and those with NAFLD in the absence of cirrhosis were not found to sufficiently benefit from routine HCC screening. For these groups, future approaches could rely on biomarker development or risk-stratification tools [34].

Similarly, mitigating high-risk alcohol consumption, especially in vulnerable populations, could theoretically reduce HCC incidence. Targeted screening, early intervention, and broader access to treatment for alcohol use disorder offer practical interventions.

Actionable Prevention Strategies

To reduce HCC incidence on a population level, public health efforts should emphasize the following:

o Hepatitis B and C management: hepatitis screening, early treatment, outreach to vulnerable populations, hepatitis-specific care teams, and blood bank safety protocols [35].

o Risk-based surveillance: education/promotion of HCC screening in at-risk populations, such as those with cirrhosis or chronic hepatitis B, particularly as they age.

o Obesity prevention and treatment: healthful food access, regular physical activity, and related medical interventions when appropriate [36].

o Alcohol use reduction: systematic identification of high-risk alcohol use coupled with community education efforts and referrals for treatment [37].

o Needle exchange and harm reduction: safe needles to reduce potential harm in individuals with intravenous drug exposure [35].

Note that the various domains and their corresponding actions are intentionally broad in scope. Some of the recommendations are underpinned by evidence-based research and established guidelines, such as those pertaining to HCC surveillance in individuals at heightened risk. Conversely, other suggestions, such as those related to blood bank safety protocols, have not been extensively studied in the context of hepatitis transmission or HCC incidence and are instead grounded in general knowledge and practical evidence.

Future research opportunities

Several important questions remain. First, our study did not examine HCC incidence by socioeconomic status or education level. Given that the SEER database reflects the national population in these metrics, future work should more definitively explore how these social determinants affect HCC incidence and outcomes. As previously noted, our paper found a similar declining incidence of HCC based upon SEER data. Although a useful database, confirmation of this trend by analyzing a different database would further strengthen our claims.

Additionally, our data ends in 2020. Changes in physical and mental health, with decreased physical activity and worsening mental health issues, were observed during the pandemic [38]. With the COVID-19 pandemic disrupting healthcare access, social behaviors, and chronic disease management, it is critical to analyze trends post-2020. Theoretically, delayed screening or medical care could influence HCC incidence and outcomes in the following years. It would be helpful to compare HCC incidence and HCC screening rates in applicable individuals during 2020 to 2025. This would enable us to determine if a continued decline in HCC incidence in those years was real or artifactual in the setting of decreased screening and medical care, in general. If reliable data, such as from a national database, cannot be found, one could evaluate the volume of liver clinic visits as a surrogate marker for patients receiving recommended care for chronic liver disease during these years. This would require comparisons to pre-COVID volumes as a control group, while also potentially excluding data from the earliest months of the pandemic when people were advised to remain home and socially isolated.

Emerging trends in liver cancer, particularly the increasing incidence of ICC, warrant hypothesis-driven investigation into the role of epigenetic differences and/or environmental exposures across generations. We hypothesize that factors, which may differ across generations, interact with molecular pathways to drive shifts in liver cancer epidemiology. This hypothesis is, in part, based upon the notable rise in early-onset colorectal cancer (CRC), which is thought to reflect broader generational changes, such as increasingly sedentary lifestyles, childhood exposure to antibiotics with downstream effects on the gut microbiome, and a higher prevalence of sporadic gene mutations [39]. These observations in CRC support the need to explore whether similar mechanisms may contribute to current liver cancer trends. To test these hypotheses, a longitudinal, multi-cohort epidemiologic study incorporating molecular profiling and detailed environmental exposure histories would be helpful.

Finally, while our data focused on incidence trends, future work should investigate survival outcomes. Preliminary data suggests that higher socioeconomic status is associated with better HCC survival [40]. Further research could help identify the specific factors, such as healthcare access, insurance coverage, or treatment facility type, that contribute to improved prognosis.

## Conclusions

In conclusion, early diagnosis through better screening measures, together with patient and physician education on up-to-date guidelines and treatment protocols, should continue to be a priority to improve patient outcomes. As discussed, not all types of liver cancer are declining, which underscores a need for more nuanced cancer-specific strategies. HCC diagnosis and management have evolved in the United States, with a more optimistic outlook regarding incidence trends. Disparities exist among demographic groups, as highlighted in our results. Males and older adults continue to experience the highest incidence rates. Interventions to decrease unhealthy alcohol use among males and the burden of chronic hepatitis among older adults would be expected to reduce these disparities. HCC incidence climbed over the past two decades, with a decline beginning only in recent years. Most groups demonstrated some degree of decline, although variable, beginning around 2015. The reason for the decline is multifactorial and, as examined in this paper, somewhat nuanced based upon an individual’s demographics and/or social factors. The burden or incidence of HCC remains higher than at the start of this study. 

Therefore, continued interventions are needed to address the underlying risk factors of HCC. Declining HCC incidence is suggestive of improved management and/or prevention of chronic liver diseases in recent years. The actionable prevention strategies, noted in our discussion, can help and should be provided in an equitable fashion. This includes evidence-based screening and surveillance patterns. The American Association for the Study of Liver Diseases surveillance guidelines recommend ultrasound surveillance with or without alpha-fetoprotein testing on a semi-annual basis in anyone with cirrhosis because of demonstrated improvements in survival. Finally, this paper’s examination of incidence trends reveals that certain targeted interventions could reduce disparities in HCC incidence among various groups. For example, an increased focus on screening for chronic hepatitis among older adults would decrease age-based disparities. Further, a multifaceted approach, ranging from public health campaigns against alcohol abuse and clinical interventions for individuals with alcohol use disorder, would be expected to diminish some sex-based disparities that exist in HCC incidence. Closing racial gaps is likely more complex, and culturally sensitive approaches to address these disparities are an area for further work. Regardless, across all populations, the most effective path forward lies in strengthening prevention, early detection, and treatment efforts to reduce the national burden of HCC and sustain the downward trend in its incidence.
